# Toward Contactless Biology: Acoustophoretic DNA Transfection

**DOI:** 10.1038/srep20023

**Published:** 2016-02-01

**Authors:** Thomas Vasileiou, Daniele Foresti, Adem Bayram, Dimos Poulikakos, Aldo Ferrari

**Affiliations:** 1 Laboratory of Thermodynamics in Emerging Technologies, Department of Mechanical and Process Engineering, ETH Zurich, Sonneggstrasse 3, CH-8092 Zurich, Switzerland; 2 Harvard University, School of Engineering and Applied Sciences, Wyss Institute for Biologically Inspired Engineering, Northwest Labs, B146.40, 52 Oxford Street, Cambridge, MA 02138, USA

## Abstract

Acoustophoresis revolutionized the field of container-less manipulation of liquids and solids by enabling mixing procedures which avoid contamination and loss of reagents due to the contact with the support. While its applications to chemistry and engineering are straightforward, additional developments are needed to obtain reliable biological protocols in a contactless environment. Here, we provide a first, fundamental step towards biological reactions in air by demonstrating the acoustophoretic DNA transfection of mammalian cells. We developed an original acoustophoretic design capable of levitating, moving and mixing biological suspensions of living mammalians cells and of DNA plasmids. The precise and sequential delivery of the mixed solutions into tissue culture plates is actuated by a novel mechanism based on the controlled actuation of the acoustophoretic force. The viability of the contactless procedure is tested using a cellular model sensitive to small perturbation of neuronal differentiation pathways. Additionally, the efficiency of the transfection procedure is compared to standard, container-based methods for both single and double DNA transfection and for different cell types including adherent growing HeLa cancer cells, and low adhesion neuron-like PC12 cells. In all, this work provides a proof of principle which paves the way to the development of high-throughput acoustophoretic biological reactors.

Acoustophoresis has recently emerged as a potentially powerful technology to operate contactless manipulations of liquids and solids in air^1^,^2^,^3^. Previous approaches were able to demonstrate the concept of acoustic levitation as a containerless support of samples and its targeting biological applications through the analysis of red blood cells^4^ and the handling of zebrafish embryos^5^. However, the controlled spatial movement of small volumes of levitated solid and/or liquid matter in air with the help of acoustophoresis^6^,^7^ was only recently demonstrated. One of the major novelties of the acoustophoretic motion of acoustically levitated matter is therefore the ability to not only levitate droplets of solutions (as in acoustic traps) but realize their controlled planar motion, bring them to contact, and induce their mixing. We have previously demonstrated the applicability of this process to chemical reactions and to biochemical processes^6^ which can be fully realized in air with the main advantage of a superior control over the solute concentration, the avoidance of contamination from the container, and the minimization of reagent loss through contact with the support materials. Additionally, the acoustic streaming induces vortices inside the levitated droplet, enhancing the mixing^8^.

The application of this methodology to molecular and cellular biology promises to significantly reduce the use of reagents and would allow for new categories of biological substrate-free studies. Yet, several hurdles remain to be addressed; firstly, the injection and ejection in and from the acoustophoretic handling system have to be carefully designed, in order allow precise manipulation of small volumes. Additionally, especially when dealing with microliter and sub-microliter volumes, precise control of the environmental conditions is crucial. The small volumes (0.1–10 μl) used in acoustic levitation limit the residence time of liquid samples due to evaporation which may change the solute concentration in the medium. Moreover, potential contamination of the levitated sample through the air must be considered.

Cell viability is a key aspect in building a reliable biological platform. In acoustophoresis, sample manipulation and suspension are achieved by means of ultrasound acoustic waves. Previous studies showed that ultrasounds in a wide range of frequencies interact with cells and cell membrane disruption can occur depending on the ultrasound intensity and exposure time^9^,^10^. In fact sonoporation, a technique used to promote the internalization of drugs or nucleotides into cells, is based on the controlled membrane permeabilization by high^11^ or low intensity ultrasound^12^. Although in the case of acoustophoresis in air the acoustic power transmitted from the surrounding medium to the sample is relatively low (of the order of 0.1% for a water droplet in air) due to orders of magnitude difference in acoustic impedances, there are currently no detailed studies investigating the effect of this treatment on mammalian cells. A typical indicator of cell viability is the study of the cytotoxicity of chemical or physical treatments of eukaryotic cells, generally assessed via commercial, live or dead assays^13^,^14^. These tests are based on the cell permeation of fluorescent compounds signaling the alteration of the membrane structure, which takes place in the early phases of apoptosis^14^. While these assays reliably report on the viability of a manipulation procedure, they fail to detect more subtle levels of toxicity interfering with the cell metabolism, differentiation and thus functionality.

DNA transfection is a very common procedure in modern cell biology and is fundamental both to biomedical applications^15^ and to basic biological research^16^. In the latter case, constructs for the expression of fusion proteins coupled with a fluorescent reporter are introduced in cells under the control of constitutive promoters^17^. The efficiency of DNA transfection varies largely depending on the cell type and on the molecular weight of the induced protein^17^,^18^. In particular, transformed adherent cells (such as cancer cell lines) are normally transfected with high efficiency for the expression of small proteins or peptides^19^. Cells growing with low adhesion to the substrate and having neuronal phenotype, require substantially more complex procedures and yield lower transfection efficiency^20^,^21^. Similarly, the use of DNA plasmids encoding for large chimeric proteins generally correlates with a reduced transfection efficiency^22^. A great number of competing technologies are commercially available for transfection, each specifically optimized for one application or cell type^23^. A broad classification can be made into liposome and liposome-free procedures, each having specific advantages in terms of efficiency, cell viability, and time to protein expression after transfection^15^. In all cases, transfection is obtained by mixing or adding a solution containing DNA and chemical reagents (i.e. liposome, salts, etc.) to a cell culture either in suspension or growing on a plastic support. Handling and preparation of reagents require multiple passages in contact with laboratory plastic-ware, each representing a potential source of contamination and contributing to a substantial loss of reagents.

In order to establish acoustophoresis as an alternative technique for biological protocols, here we design, experimentally verify and implement a novel approach, combining conventional reservoirs (both sources and sink) and acoustophoretic contactless handling in a semi-automated system. With this new system, we provide here the first thorough investigation of cytotoxicity in eukaryotic cells with various acoustophoretic levitation procedures. We detected potential small metabolic perturbations based on the controlled differentiation of PC12 cells induced by nerve growth factor (NGF)^24^,^25^. Interference with the complex neuronal differentiation pathway elicited by NGF binding to its receptors results in a measurable reduction of both the number and the length of neurites produced by stimulated cells. Finally, we selected a commercially available, liposome-free tool for DNA transfection and compared the efficiency of a standard transfection protocol with a procedure using the same reagents but optimized for our contact-free acoustophoretic setup. The comparison was performed using single or double DNA transfection and applied to small or large DNA complexes, to high or low adhesion cells and to cancer or neuronal cell lines.

## Results

### Acoustophoretic platform for biological applications

**Figure 1:**
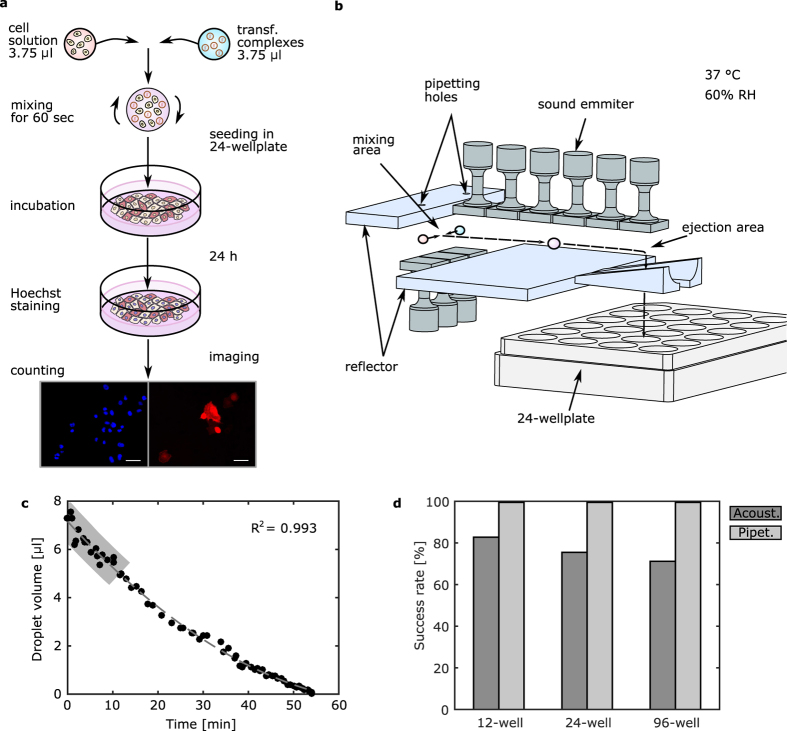
Acoustophoretic DNA transfection: (**a**) Illustration of the experimental protocol. Cell suspension solution and fluorescent protein encoding plasmid with transfection reagent in buffer are prepared at specific concentrations. A 3.75 μl droplet of cell solution is merged with a droplet of same volume containing the transfection complexes and it remains levitated in the platform for 60 sec. After ejection, the cell and transfection complex mixture is transferred in well plates and kept in the incubator. After 24 h, the nuclei are stained with Hoechst and the cells are imaged in the microscope. The transfection efficiency is calculated by counting the total number of cells (Hoechst stain) and the number of cells that expressed the fluorescent protein. (**b**) Illustration of the acoustophoretic setup. The two droplets are introduced through the pipetting holes in the mixing area and merged in the middle. The merged droplet is transferred to the ejection area, where it is placed into the well underneath and a motorized 2-axes linear stage moves the neighbouring well below the ejection area to receive the next mixture droplet. (**c**) Time evolution of the volume of a levitated water droplet with initial volume of 7 μl at 37 °C and 60% relative humidity. Highlighted area marks the interval where the volume reduction is less than 30% of the initial volume. (**d**) Success rate of a DNA transfection experiment from injection to ejection in the well with comparison to the success rate of standard pipetting, for different well plate sizes.

A broad palette of techniques for cell manipulation are based on the mixing of cell suspensions with chemical substances^14^, macromolecules^16^ and/or nanoparticles^25^. The DNA transfection of suspended mammalian cells follows the same pattern. The cell suspension used here is mixed with a solution of transfection complexes (DNA and chemicals) before plating (Fig. 1a). A similar protocol is therefore applicable both in a standard culture container and in a contactless acoustophoretic configuration.

The design of the setup for contactless manipulation was based on the acoustophoretic concept of discretized sound emitting elements operating with a time-depended velocity amplitude^6^. In this case, the emitting elements were arranged in a T-shape configuration as shown in Fig. 1b. The T-shape allowed the mixing of two samples at a minimal transport distance. Injection was achieved simultaneously at two distinct points through pipetting holes located at the up-facing emitter part of the device. The mixing of the samples took place in the same area. Serial adding of droplets of different solutions can be achieved by moving the merged droplet in the initial injection position and repeating the merging procedure. A distance of 0.5283 times the wavelength between the reflector and emitters was found to permit a smooth merging of the droplets during mixing. To allow for the injection and ejection of the liquid samples, the sound emitters operated both at the top and bottom of the levitation plane (Supplementary Video S1). Emitters operating at different planes were excited with a phase difference of 180^o^ whereas those in the same plane were excited in phase, such as the interference of the acoustic waves acted in a constructive manner.

The issue of the contactless ejection of acoustophoretically levitated samples, critical to any device, has not been study to date. The literature regarding conventional acoustic levitators only reports manual extraction though needles or nozzles^3^,^26^,^27^. Since the acoustophoretic forces are of the same order of magnitude as the gravity force^28^, the latter was selected to perform the ejection out of the acoustophoretic system. Additionally, the gravity force has a constant direction and magnitude per unit mass, simplifying the design of the system. Indeed, the ejection procedure can be facilitated by simply acting on the magnitude of the acoustophoretic forces: the free fall of the sample is obtained by reducing the power of a specific set of transducers (Fig. 1b).

**Figure 2:**
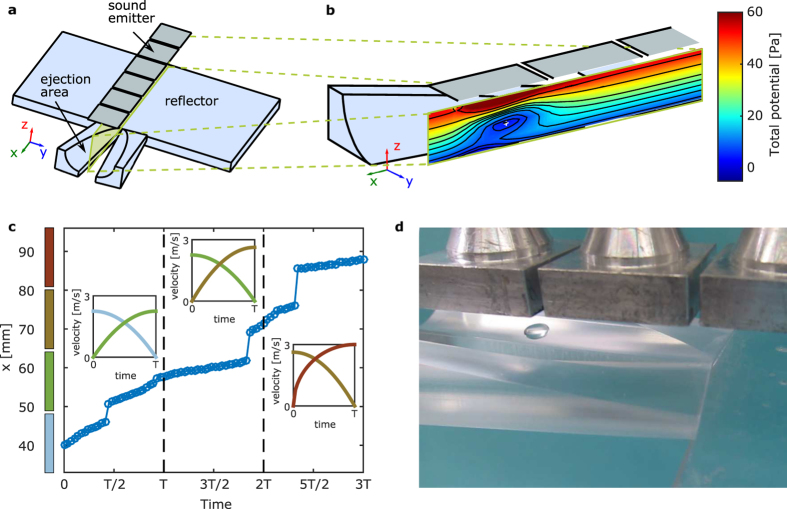
Acoustophoretic ejection concept: (**a**) Illustration of the ejecting reflector. The flat part of the reflecting surface is used to transport the sample above the specially designed V-shape part of the reflector. There the sample can be suspended above a container and when the acoustic power is reduced, gravity drives it into the container. The ejecting part has a cylindrical shape to enhance the acoustic field through focusing. A progressive widening of the spacing facilitates the smooth transition from the flat region to the ejection point. (**b**) Total potential at the ejection point, when only the last emitting element is operating (*V*_0_ = 3 m/s, H = 0.5639 λ, see SI). Levitation of the sample is expected at the potential minimum, indicated by a white cross in the figure. (**c**) Simulation of the sample position during transport from the flat part to the ejection point (x = 88 mm). Three transitions with period T over four sound emitting elements are shown in the figure. The position of the sound emitting elements is indicated next to the vertical axis by the coloured rectangles. The vibration velocity amplitude ramping of the emitting elements is plotted in the insets above each transition. (**d**) Droplet of water levitated above the ejection point.

The ejection system implementation employed an inverted levitator configuration, reflector at the bottom and transducers on top, and a modified reflector design (Fig. 2a). The reflector comprised of a flat area for transporting the sample at the ejection point and a V-shaped region with an opening gap to allow free fall when the corresponding set of emitters were turned off. For a stable and smooth sample handling, the opening was designed as a triangular geometry with a progressive notch. A cylindrical shape was given to the two parts of the reflector enhancing the acoustic field through focusing of the acoustic waves. Simulation of the total potential (see SI) revealed that suspension of samples above the notch is possible (Fig. 2b,c), if the acoustic power is high enough (emitter vibration velocity around 3 m/s). Transport of the samples in the ejection point was facilitated by the progressive widening of the gap and an increase of the acoustic power of the emitters closer to the ejection point (Fig. 2c and SI). The distance of 0.5639 times the wavelength between the emitters and the flat part of the ejecting reflector was found to give smooth transport and adequate levitation force at the ejection point. The ejecting reflector was kept at the same plane as the emitters facing up to allow for a transition between the mixing and ejection area of the setup. The entire process including the ejection procedure is captured in Supplementary Video S1.

The acoustophoretic system was designed to interface with a typical biochemical container – the cell culture multi-well plates. Nevertheless, the ejection of the samples was taking place at a single exit point. To fill the different wells of the plate, a fully automatized, two axe linear stage system was selected (NRT 100/M-NRT 150/M, Thorlabs Inc) to work under the environmental condition of interest (max. velocity of 30 mm/s, max. humidity 70%, max. temperature of 40^o^C). The procedure of injecting two 3.75 μl droplets, merging, mixing for 60 s and delivering them into a well was tested for different well-sizes in commercial multi-well culture plates. Figure 1d shows the success rate of the full procedure, ranging from more than 80% for 12-well plates to 70% when 96-well plates were used.

The entire setup was enclosed in a transparent Plexiglas box, which physically isolated the handling and storing of samples from the external environment, minimizing the risk of contamination. The temperature and humidity inside the enclosure were kept at 37 ± 0.2 °C and 60 ± 2% respectively. To assess the evaporation rate of the levitated liquid samples in these conditions, a 7 μl water droplet was introduced in the acoustophoretic device and left levitating until full evaporation. Images of the droplet were taken at every minute and the droplet volume was extracted for the analysis. For a droplet of this size, a residence time of 10 min reduced the volume by less than 30%. This amount can be assumed viable with respect to cell manipulation (Fig. 1c). Overall, these results demonstrate the capability of the developed system to actuate the contactless handling of the cell specimens in an acoustophoretic DNA transfection protocol.

### Cytotoxicity

While conventional viability assays are based on the early detection of cell apoptosis induced by physical or chemical toxic agents, we aimed at evaluating the potential interference of acoustophoretic handling with more specialized molecular activities, such as cell differentiation and polarization. To do so we monitored the effect of acoustic levitation on the neuronal differentiation of PC12 cells upon stimulation with nerve growth factor (NGF)^29^,^30^. Unstimulated PC12 cells can grow in culture as a conventional low-adhesion cell line. Stimulation of the neuronal differentiation pathway induces a growth arrest and initiates a process leading to the generation of cell neurites, which can be detected in optical microscopy as cylindrical protrusions of several tens of microns originating from the cell body^29^,^30^. The generation of such neurites takes normally place in few days upon NGF stimulation, and importantly, it is easily compromised by cytotoxic agents causing even small metabolic perturbations of the cell.

In our experimental set up we tested the cytotoxicity of acoustophoretic levitation and transfection using a plasmid encoding for a microtubule binding fluorescent construct, EMTB-GFP^31^ on PC12 cells. Transfection was induced using the liposome-free commercial transfection kit JetPRIME. In the acoustophoretic setup, PC12 cells were suspended and mixed with a single DNA transfection complex, or with induction medium for two test residence times of 1 or 5 min. The same procedure was repeated using a sterile plastic container in a conventional protocol operated within a biological flow cabinet.

**Figure 3:**
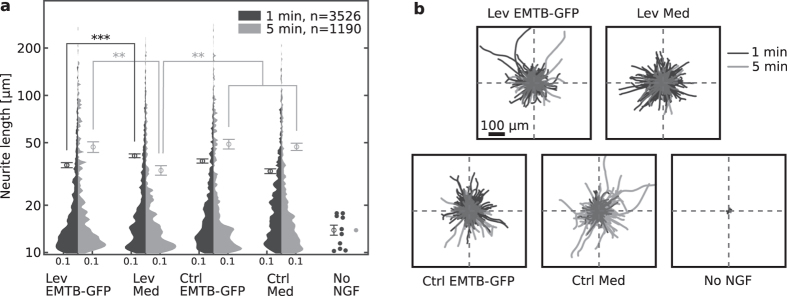
Biocompatibility of the acoustophoretic mixing platform: (**a**) Estimated probability density and mean neurite length grown by PC12 cells after 6 days in culture. For cases with not sufficient data, the individual measurements are plotted instead. In the five tested conditions the cells were mixed in the acoustophoretic platform with a transfection complexes (Lev EMTB-GFP) or with induction medium (Lev Med), in commercial tissue culture plastic containers with transfection complexes (Ctrl EMTB-GFP) or with induction medium (Ctrl Med) or plated directly in wells containing induction medium without NGF. Significant differences are indicated by asterisks (** for p < 0.01 and *** for p < 0.001). Case without NGF is excluded from significance testing due to inadequate sample size. The open circles indicate the mean value and the error bars represent the standard error of the mean. The horizontal axis indicates probability density and the histograms are normalized such that equal width corresponds to equal probability. The neurite length scale is logarithmic for better inspection. Total number of measured neurite is shown at the figure legend for each residence time. (**b**) Tracing of the neurites corresponding to the five cases described before, showing the directionality and the length. Neurites for residence time of 1 min are depicted in black, whereas for residence time of 5 min in grey.

Figure 3a shows the average length and the probability density function (PDF) of neurites generated by PC12 cells upon stimulation with NGF for 6 days. The neurite lengths in the unstimulated control samples are shown in the same figure as single points, since the total number of detectable neurites was too small for estimating the PDF. Neuronal differentiation upon stimulation with NGF was always substantial compared to the basal unstimulated cells, both with respect to the mean length and the total number of neurites.

The neurite length measured for stimulated cells which were subjected to acoustophoretic manipulation was generally similar to the values measured in non-levitated samples (Fig. 3). A small but significant decrease was detected for cells levitated for 5 min and mixed with induction medium. However, this variation was within the fluctuation observed in control samples and therefore probably due the presence of a higher number of small neurites (Fig. 3a).

The effect of the resident time in the acoustophoresis set-up was also negligible. No clear trend in neurite length was detectable between the cell treated with a residence time of 1 or 5 min. Since the statistical analysis revealed no considerable difference between any other combinations of groups, the punctual variations are attributed to difference in the PDF shape rather than to signs of cytotoxicity. Figure 3b provides a visual representation of the lengths and orientation of neurites. Overall, these data demonstrate that no acoustophoretic-related cytotoxicity could be detected upon NGF-induced neuronal differentiation of PC12 cells. Longer mixing times up to 5 min do not represent a stress factor for the cells.

### DNA Transfection

DNA transfection is widely used in biological applications and a number of commercially-available chemical-based methods rely on adding the transfection complexes solution to a subconfluent cell monolayer. The procedure typically requires one day preparation for the seeding and the adhesion of cells to the substrate. In alternative transfection protocols^32^,^33^, the transfection complexes are either placed in the culture support before cell seeding or are directly mixed with the solution of suspended cell prior to seeding. Upon seeding and adhesion, cells start to express the induced recombinant proteins. In the case of fluorescent proteins, the efficiency of transfection can be revealed by determining the fraction of cells emitting a fluorescent signal.

For the sake of our tests we adopted a transfection procedure based on the mixing of cells and transfection reagents prior to seeding. In our approach a 3.75 μl droplet of cell solution was merged with an equal volume of transfection complexes solution. In the acoustophoretic setup, the merged droplet was mixed for 1 min and then plated in a well of a multi-well cell culture plate already containing fresh, complete medium. The transfection efficiency was assessed at 24 h after transfection (Fig. 1a).

Specifically, the contactless mixing approach was tested on both an adherent cancer cell (HeLa) and low-adhesion neuronal-like cell (PC12) line. For single DNA transfection tests a plasmid encoding the Histon H2B – Green Fluorescent Protein (H2B-GFP) chimeric construct was used. The transfection reagent was obtained from the jetPRIME commercial kit. Our choice was based on its low toxicity and the well-established use in single or multiple plasmid transfection^34^,^35^.

**Figure 4:**
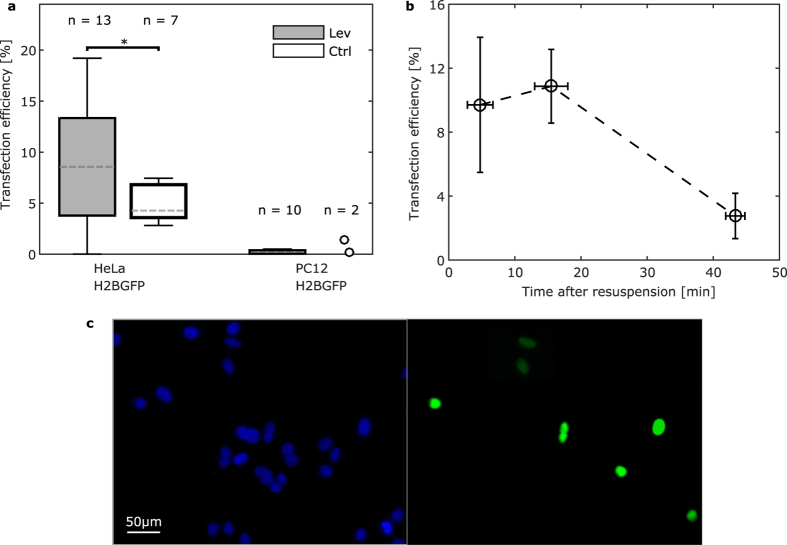
Efficiency for single plasmid transfection: (**a**) Comparison of the transfection efficiency for adherent (HeLa) and suspension cell (PC12) lines with the H2B-GFP plasmid using contactless mixing in the levitator and mixing in commercial tissue culture plastic containers. Cell solution concentration was kept at 4000 cells/μl for all the experiments and both cell lines. Significant differences are indicated by asterisks (* for p < 0.05). Sample size is indicated above each population. Box spans from the 1^st^ to 3^rd^ quartile with Tukey style whiskers. Dashed lines indicate the medians. (**b**) Dependency on the time elapsed from re-suspending HeLa cells to the mixing in the acoustophoretic device on the transfection efficiency. Measurements from two independent experiments were merged and averaged over 10 min intervals. Points represent the mean and error bars the standard error of the mean. (**c**) Microscope fluorescent images of the nucleus staining (Hoechst) and the H2B-GFP protein for HeLa cells.

The results of acoustophoretic transfection were compared to those obtained applying the same procedure in a standard protocol using commercial tissue culture plastic containers. The single DNA transfection efficiency of the two methods for the two cell lines is reported on Fig. 4. Single DNA acoustophoresis-based transfection yielded a significant increase in the transfection efficiency as compared to the container-based transfection in HeLa cells (Fig. 4a). Importantly, the transfection efficiency had a higher variability when performed in the acoustophoretic setup, due to the dependence of the transfection efficiency on the residence time of cells in suspension before mixing and seeding. Since the custom-built set up could only operate one mixing event at a time, lower transfection efficiency was obtained by cells that were kept for more than 20 min in media suspension (Fig. 4b). For the case of PC12 cells the transfection efficiency was generally low, and therefore no significant variations were detectable between the two transfection approaches.

**Figure 5:**
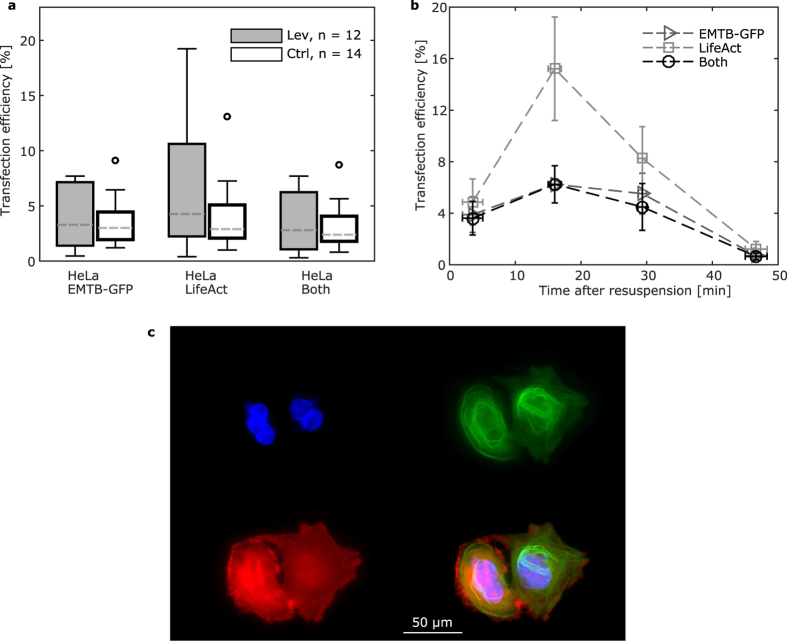
Efficiency for double plasmid transfection: (**a**) Comparison of the transfection efficiency for HeLa with the EMTB-GFP and the actin LifeAct-mCherry plasmids using contactless mixing in the levitator and mixing in commercial tissue culture plastic containers. The transfection efficiency was measured for cells expressed either of the plasmids (HeLa EMTB-GFP and HeLa LifeAct) and both of them (HeLa Both). The cell solution concentration was 4000 cells/μl for all experiments. Sample size is indicated in the figure legend. Box spans from the 1^st^ to 3^rd^ quartile with Tukey style whiskers. Dashed lines indicate the medians. Outliers are indicated with an open circle. (**b**) Dependency on the time elapsed from re-suspending cells to the mixing in the acoustophoretic device on the transfection efficiency for both plasmids. Measurements from two independent experiments were merged and averaged over 10 min intervals. Different markers show different plasmid expression. Points represent the mean and error bars the standard error of the mean. (**c**) Microscope fluorescent images for double transfected cells. The cell nuclei, tubulin and actin structures and a merged image are shown from top left to bottom right.

Additionally, we investigated the efficiency of a double DNA transfection on HeLa cells (Fig. 5). Here, we selected commercial DNA constructs, which are commonly used to visualize the two main components of the cytoskeleton, the microtubules (EMTB-GFP) and the actin filaments (LifeAct-mCherry). The transfection efficiency was not influenced by the molecular weight of the chimeric construct induced (Figs 4 and 5) and was similar for all transfected plasmids. In this case only the transfection of LifeAct yielded higher efficiency in the acoustophoretic set up, but altogether no clear difference could be measured. A more thorough analysis of the dependency of transfection efficiency on the time after resuspension of transfected cells (Fig. 5b) indicates that the best results were obtained between 10 and 20 min after resuspension, while the efficiency linearly dropped thereafter.

Altogether these data demonstrate that in addition to its contactless nature, the acoustophoresis set-up yields comparable or improved transfection efficiency as compared to standard approaches in both single and double DNA transfection protocols. The absolute efficiency values depend on the cell type and on the plasmid used and seem to strongly depend on the time after cells resuspension.

## Discussion

Acoustophoresis has the potential to open new venues in operating standard biological procedures in a contactless and controlled experimental environment. As a first step in this direction, here we provide basic experimental proof on the application of acoustophoretic handling in molecular biology.

We revisited the classic protocol for DNA transfection of mammalian cells and established an acoustophoretic mixing of cells and DNA. The relevance of the data reported is twofold. First, we demonstrate that acoustophoretic cell handling and transfection are fully viable. The ability of cells to differentiate and polarize in response to biological stimuli is fully retained upon levitation, and comparable to the results obtained in standard, container-based protocols. Second, we prove that the controlled mixing obtained by acoustophoretic stirring enhances DNA transfection efficiency when compared to similar procedures based on pipette mixing. The reason can be found in the continuous and complete mixing actuated by acoustic forces on liquids, which likely yield a more homogenous distribution of DNA transfection complexes, especially in the cases where cells are suspended in high densities.

However, higher transfection efficiency can be obtained by mixing DNA and chemical reagents and directly adding them to adherent cells previously plated on cell culture substrates^36^. In the present work, the transfection protocol optimized for adherent cells was adapted to be performed in suspension in order to obtain a complete biological reaction in air, which would have not been possible otherwise. The reduced transfection efficiency can therefore be explained by the fact that suspended cells tend to form clusters and are less exposed to the surrounding DNA-containing solution. The enhanced mixing obtained through acoustic levitation may partially counteract this effect thus improving DNA permeation of the membranes. Further studies in this direction shall optimize the transfection procedure toward the requirements of the acoustophoretic approach to yield higher transfection efficiencies. This shall include the design of dedicated chemical reagents and of improved mixing protocols.

Altogether these data pave the way to the future optimization that will develop acoustophoretic devices for the parallel and high-throughput transfection of single or multiple DNA with high efficiency and/or minimal amounts of DNA and transfection reagents. Similar protocols may be established for more complex procedures, where multiple mixing steps are necessary, and in which the capabilities of acoustophoretic handling may prove even more advantageous as compared to standard, container-based manipulations.

## Methods

### Cell culture

HeLa cells were grown in DMEM supplemented with 5% fetal bovine serum (FBS), 2 mM L-glutamine, 100 U/ml penicillin and 100 μg/ml streptomycin (all from Sigma-Aldrich, USA) at 37 °C and 5% CO_2_ prior to the transfection experiments.

PC12 cells were cultured in RPMI-1640 medium supplemented with 10% horse serum (HS), 5% FBS, 2 mM L-glutamine, 100 U/ml penicillin and 100 μg/ml streptomycin (all from Sigma-Aldrich, USA). PC12 were maintained at 37 °C and 5% CO_2_ prior to the transfection and cytotoxicity experiments.

### DNA transfection

For all transfection experiments the JetPrime transfection reagent – buffer solution combination (Polyplus-transfection SA, France) was used. The plasmids – Histone H2B-GFP, LifeAct-mCherry and EMTB-GFP – were a kind gift of Ewa Paluch (Laboratory for Molecular Cell Biology, UC London, England).

The plasmids were diluted in jetPrime buffer at concentration 2.64% w/v and the solution was mixed by vortexing. The transfection reagent was added to the solution to a final concentration of 5.01% v/v. The solution was mixed for 10 sec by vortexing and incubated for 10 min in room temperature.

For the acoustophoretic transfection of HeLa cells, the cells were initially detached from the culture plates by washing with PBS and then treating with 1x trypsin for 3 min at 37 °C. Cells were counted, centrifuged at 800 rpm for 7 min and resuspended in the fully supplemented CO_2_ independent medium at concentration of 4000 cells/μl. The 24-well plates for seeding the transfected cells were filled with 0.5 ml per well of fully supplemented CO_2_ independent medium, before the beginning of the experiment.

For PC12 suspension cells, the cells were separated to single cells by repeatedly pipetting through narrow ended tip. Cells were counted, centrifuged at 800 rpm for 7 min and resuspended in fresh culture medium at concentration of 4000 cells/μl. The 24-well plates for seeding the transfected cells were filled with 0.5 ml per well of culture medium, before the beginning of the experiment.

One 3.75 μl droplet of the cell solution and a 3.75 μl of the transfection complex solution were merged and mixed for 60 sec. Motorized pipettes (Pipetman P10M, Gilson Inc, USA) were used for increased pipetting accuracy and repeatability. The mixing of the droplets was performed both contactless using the acoustophoretic setup and in commercial tissue culture plastic containers, under a laminar flow hood. The mixed droplet was placed in 24-well plates and kept in 37 °C and 5% CO_2_ environment. After 24 h, the cells were fixed with 3% PFA solution, washed 3 times with PBS and preserved in 0.5 ml of PBS with 100 ng/μl Hoechst 33342 DNA stain.

### Acoustophoretic setup

The acoustophoretic chamber consists of the sound emitters, Langevin type piezoelectric transducers designed and manufactured in house with cross-section of 20 x 15 mm, and 5 mm thickness Plexiglas reflectors. For this size of the emitters, a spatial resolution of 400 μm has been reported^6^. A size comparison between the droplet and the emitters can be seen in Supplementary Fig. S1. The emitters are excited with a 24.24 kHz variable amplitude sinusoidal signal, composed by a custom made electronic box which is controlled by a desktop PC with Simulink Real-Time Workshop (Mathworks Inc., USA). The signal is amplified through a 3-channel voltage amplifier (PA93, APEX Microtechnology, USA) to a range of 0 – 135 V. The electronic box implements additionally a multiplexing function between the emitters and the amplifier channels.

The setup is enclosed in a double-wall Plexiglas box, fitted with 8 electric heating coils (20W-24 VDC, Thermo Technologies, France). The temperature and the humidity in the setup are measured with a digital sensor (HYT 221, IST AG, Switzerland). The temperature can be regulated by changing the mean power to the heating coils. Air stream at 350 mbar overpressure is bubbled through a temperature regulated water bath and near saturation air humidifies the chamber, while the temperature of the bath adjusts the humidity level. The environmental conditions are constantly supervised and controlled by a micro-controller (Industruino Proto, BTL cvba, Belgium).

### Cytotoxicity

For the cytotoxicity assay, the bottom of commercial 12-well cell culture plates was replaced by microscope glass slides and the well were sterilized using 70% ethanol overnight. The plates were washed 3 times with PBS (5 min each) and then incubated with 1 ml poly-L-lysine (PLL) 0.01% solution (Sigma-Aldrich, USA) per well for 5 min at 37 °C. The excess PLL solution was then removed and the wells were left to dry overnight. The plates were finally washed 3 times with PBS (5 min each) and rinsed with water.

The EMTB-GFP plasmid was mixed with the transfection reagent following the transfection protocol concentration and procedure. Low passage PC12 were gently detached from subconfluent cultures and single cells were obtained cells by repeatedly pipetting through narrow ended tip. Cells were counted and resuspended in induction medium (RPMI medium supplemented with 2% FBS, 2 mM L-glutamine, 100 U/ml penicillin and 100 μg/ml streptomycin) at a final concentration of 4000 cells/μl. The well plates were filled with 1 ml of induction medium containing NGF at a concentration of 100 ng/ml.

A 3.75 μl droplet of the cell solution merged in the acoustophoretic device with a 3.75 μl droplet of either the transfection complexes solution or induction medium. The merged droplet was then ejected to the well-plate. The same procedure was repeated in commercial tissue culture plastic containers. Further more, a 3.75 μl droplet was directly seeded in a well plate containing induction medium without NGF. Two residence times, of 1 min and of 5 min, were tested in the acoustophoretic mixing device and on plastic containers. The cells were kept in 37 °C and 5% CO_2_ for 6 days and images were acquired on the 3rd and 6th day.

### Imaging

Cell images were acquired using with an inverted Nikon-Ti wide field microscope (Nikon, Japan) with an Orca R-2 CCD camera (Hamamatsu Photonics, Japan). For samples that were not fixed, an incubated chamber was used (Life imaging services, Switzerland), where the temperature, humidity and CO_2_ concentration were kept at 37 °C, 95% and 5% respectively. Images were collected with a 20x magnification, 0.45 numerical aperture long distance air objective (Plan Fluor, Nikon, Japan). For the cytotoxicity experiments, 10 images at random spots were acquired per well using the white field channel with a differential interference contrast (DIC) filter. For the transfection experiments, 6 images were collected at random spots of the well with high cell coverage, using the fluorescent DAPI filter for detecting the cell nuclei and the respectful fluorescent filter for detecting the proteins encoded by the plasmids.

For the analysis of the droplet evaporation rate, images were acquired with a CCD camera with 3.8x optical zoom (S95, Canon, USA).

### Image Analysis

For the analysis of the neurite length in NGF stimulated PC12 cells, the collected images were loaded into ImageJ (National Institute of Health, USA) and analyzed using the NeuronJ plugin^37^.

For measuring the transfection efficiency, the collected images were loaded into ImageJ and processed using the following protocol: the DAPI fluorescent channel was enhanced using the ‘Subtract Background’ filter of ImageJ and the image was converted to binary using the ‘Threshold’ tool (‘default’ thresholding method) of ImageJ. The binary image was further de-noised using a subsequent application of ‘Erode’ and ‘Dilate’ filters. Cells in close proximity where separated using the ‘Watershed’ algorithm. Cell nuclei were segmented using the ‘Analyze particle’ tool of ImageJ and exported as regions of interest. The segmentation results were overlaid on the original DAPI fluorescent image and were inspected and corrected by the user. The corrected nuclei regions were overlaid on the fluorescent image of the transfection marker and the mean intensity of each of the segmented regions were measured. The number of transfected cells was determined by counting the regions with mean intensity above a given threshold. Cells counted as transfected were also inspected and corrected by the user. Each set of images was processed by two individual users separately and the transfection efficiencies were averaged.

For the analysis of the droplet evaporation rate, images were loaded to into ImageJ and compiled into a stack. The slices alignment was corrected using the ‘Template matching’ plugin of ImageJ. An ellipse was fitted manually to the droplet outline using the ‘Oval’ tool of ImageJ and the major and minor axes were measured. The volume of the droplet was obtained under the assumption of oblate spheroid^38^, using the equation:


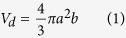


where *V*_*d*_ is the droplet volume, 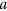 and 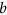 are the fitted ellipse major and minor axes, respectively.

### Statistical analysis

Statistical comparison of the transfection efficiency was performed using the non-parametric one-sided Kolmogorov-Smirnov test (α = 0.05). Statistical comparison of the neurite length in PC12 cells was performed using the non-parametric Kruskal-Wallis one-way analysis of variance test (α = 0.05), given the high skewness of the PDF’s and the low coefficient of variance heterogeneity^39^ among the groups (c = 0.212 for 1 min groups and c = 0.315 for 5 min groups). Neurite population grown without NGF treatment was excluded from the significance test due to two orders of magnitude smaller sample size than the other groups. All quantitative measurements reported are expressed as average values ± the standard error of the mean. The total number of events counted is shown either above each population or in the legend of the presented graphs.

## Additional Information

**How to cite this article**: Vasileiou, T. *et al.* Toward Contactless Biology: Acoustophoretic DNA Transfection. *Sci. Rep.* **6**, 20023; doi: 10.1038/srep20023 (2016).

## Supplementary Material

Supplementary Information

Supplementary video
