# Supplementary material for: Toward Contactless Biology: Acoustophoretic DNA Transfection
Source: Sci Rep. 2016 Feb 1;6:20023. doi: 10.1038/srep20023 (PMC4734324; doi:10.1038/srep20023)
Supplement: Supplementary Information [file srep20023-s1.pdf]

Supplementary information

Toward Contactless Biology: Acoustophoretic DNA Transfection

Thomas Vasileiou, Daniele Foresti, Adem Bayram, Dimos Poulikakos & Aldo Ferrari

Total radiation potential

The time-averaged acoustic potential acting on a small rigid sphere inside and an acoustic field has been derived in by Gor'kov[1], as:

$$\tilde{U}_a = 2\pi R_s^3 \left(\frac{p_{rms}^2}{3\rho_0 c^2} - \frac{\rho_0 v_{rms}^2}{2} \right) \quad \text{(S1)}$$

where p_{rms} and v_{rms} are the root mean square values of the acoustic pressure and the particle velocity, ρ_0 and c is the density and the speed of sound of the fluid and R_s is the radius of the sample. The equation above is valid for sample sizes that are small compared to the wavelength and when the density of the levitated sample ρ_s is much higher than the density of the surrounding medium ($R_s/\lambda < 0.1$ and $\rho_s \gg \rho_0$). In our setup, both of these assumptions are met.

In addition, the gravity exerts a force to the sample, modeled by the gravitational potential as:

$$\tilde{U}_g = \frac{4}{3}\pi R_s^3 \rho_s g z \quad \text{(S2)}$$

where $g = 9.81$ m/s^2 is gravitational acceleration and z vertical coordinate of the center of mass. The total radiation potential is given as the sum of these two components[2]

$$\tilde{U}_{tot} = \tilde{U}_a + \tilde{U}_g . \quad \text{(S3)}$$

The total potential field is proportional to the volume of the sample and it is convenient to normalize it by a volume-like factor:

$$U_{tot} = \frac{\tilde{U}_{tot}}{2\pi R_s^3} \tag{S4}$$

resulting in dimensions of pressure.

The force per unit volume acting on the sphere is calculated as by the gradient of the total potential as:

$$\vec{F} = -\nabla U_{tot} \tag{S5}$$

and the point in space where the sample can be stable levitated (levitation node) coincides with the potential minimum.

The total potential can be estimated if the acoustic pressure and particle velocities fields are known. The acoustic pressure p is calculated in the frequency domain using the linear acoustic Helmholtz equation:

$$\Delta p + k^2 p = 0 \tag{S6}$$

where $k = \omega/c$ is the wavenumber and ω is angular velocity of interest. The particle velocity can be recovered from a known pressure field as:

$$\vec{u} = -\frac{\nabla p}{j\omega\rho_0} \tag{S7}$$

where $j = \sqrt{-1}$ is the imaginary unit.

Numerical Simulations of Transitions

The position of the sample inside the acoustophoretic device during transport can be calculated using the total radiation potential, as has been already demonstrated and experimentally validated[3]. The transport of sample can be simulated numerically by changing the vibration velocity amplitude of the emitting surfaces. For each emitter velocity combination, the total potential minimum coincides with the sample position.

For a flat reflecting surface, it has been demonstrated that quadratic ramping of the subsequent emitter velocity yields smooth transitions[3]. For complex reflector geometries, the optimal curve may vary; in the case of the reflecting emitter, higher vibration velocities and modification of the ramping curves are needed to compensate for the loss of reflecting surface. In our setup, quadratic amplitude modulation of the vibration velocity was used for all emitters, except from the one directly above the ejection point, which follows a square root ramping curve as

$$V_{ej} = V_{ej,max}\sqrt{1-\left(1-\frac{t}{T}\right)^{2}} \tag{S8}$$

where $V_{ej,max}$ is the maximum emitter vibration velocity, t is the time and T is the period of the single transition.

The simulations of the process were performed using the finite element method (FEM) software COMSOL Multiphysics 5.1. The reflecting surfaces were modelled as sound hard boundaries and at the emitting surfaces a given vibration velocity was prescribed. Open boundaries were modelled by the plane wave radiation boundary condition, so acoustic waves can leave the computation domain with minimum reflection. Symmetry boundaries conditions were used at the middle plane of the ejecting part, to minimize the computational elements.

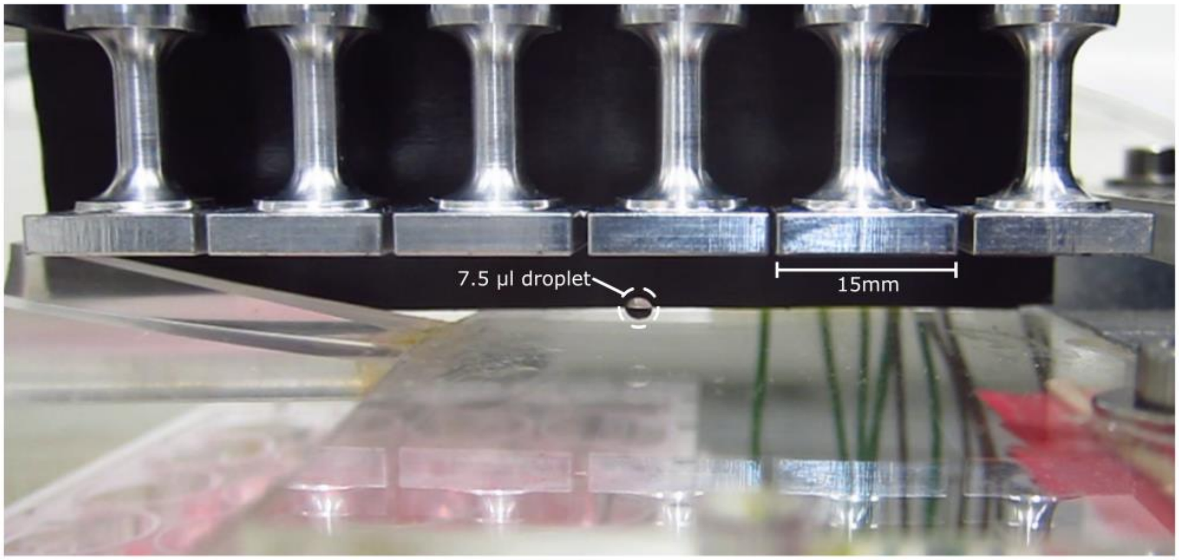

Fig S1: Comparison between the size of the sample (a 7.5 µl droplet containing living cells) and the sound emitter.

Supplementary Video S1: This video shows the DNA transfection procedure in the acoustophoretic setup.

References

1 Gorkov, L. P. Forces Acting on a Small Particle in an Acoustic Field within an Ideal Fluid. *Dokl Akad Nauk Sssr+* **140**, 88-& (1961).

2 Collas, P., Barmatz, M. & Shipley, C. Acoustic Levitation in the Presence of Gravity. *J Acoust Soc Am* **86**, 777-787, doi:Doi 10.1121/1.398200 (1989).

3 Foresti, D., Nabavi, M., Klingauf, M., Ferrari, A. & Poulikakos, D. Acoustophoretic contactless transport and handling of matter in air. *Proc Natl Acad Sci U S A* **110**, 12549-12554, doi:10.1073/pnas.1301860110 (2013).
